# Wet age related macular degeneration management and follow-up

**Published:** 2016

**Authors:** Malciolu Radu Alexandru, Nica Maria Alexandra

**Affiliations:** * Department of Ophthalmology, “Dr. Carol Davila” Central Military Emergency University Hospital, Bucharest, Romania

**Keywords:** wet age related macular degeneration, anti-VEGF, Pro Re Nata, Treat and Extend

## Abstract

Age-related macular degeneration (AMD) is referred to as the leading cause of irreversible visual loss in developed countries, with a profound effect on the quality of life. The neovascular form of AMD is characterized by the formation of subretinal choroidal neovascularization, leading to sudden and severe visual loss. Research has identified the vascular endothelial growth factor (VEGF) as an important pathophysiological component in neovascular AMD and its intraocular inhibition as one of the most efficient therapies in medicine. The introduction of anti-VEGF as a standard treatment in wet AMD has led to a great improvement in the prognosis of patients, allowing recovery and maintenance of visual function in the vast majority of cases. However, the therapeutic benefit is accompanied by a difficulty in maintaining the treatment schedule due to the increase in the amount of patients, stress of monthly assessments, as well as the associated economic burden. Therefore, treatment strategies have evolved from fixed monthly dosing, to individualized regimens, aiming for comparable results, with fewer injections. One such protocol is called “pro re nata”, or “treat and observe”. Patients are given a loading dose of 3 monthly injections, followed by an as-needed decision to treat, based on the worsening of visual acuity, clinical evidence of the disease activity on fundoscopy, or OCT evidence of retinal thickening in the presence of intra or subretinal fluid. A different regimen is called “treat and extend”, in which the interval between injections is gradually increased, once the disease stabilization is achieved. This paper aims to review the currently available anti- VEGF agents – bevacizumab, ranibizumab, aflibercept, and the aforementioned treatment strategies.

Age related macular degeneration (AMD) is referred to as the leading cause of severe, irreversible blindness in developed countries worldwide, with a profound effect on the quality of life of affected individuals, as well as on the health care systems, due to the increase of life expectancy, number of reported cases and expensive treatments [**11**]. Although 80 per cent of the patients have non-neovascular, or atrophic AMD, the neovascular form of the disease is responsible for nearly 90 per cent of the severe, central visual acuity loss associated with AMD [**1**]. The advances in the medical research have identified the Vascular Endothelial Growth Factor (VEGF) as a key pathophysiological factor in the development of neovascular AMD, with an essential role in angiogenesis, vascular permeability, and inflammatory response [**4**]. Furthermore, the innovations in the diagnostic techniques, such as Spectral Domain Optical Coherence Tomography (SD-OCT) allow high quality visualization of disease morphology, correct diagnosis, and efficient follow-up [**11**]. The introduction of anti VEGF intravitreal injections has opened a new therapeutic window in the management of wet AMD, thus efficiently blocking the pathophysiological process of AMD, with a restoration of retinal morphology and the maintenance of its function. Injections are considered safe, well tolerated, with few adverse reactions [**1**]. In the past years, anti VEGF injections have become the standard treatment for wet AMD, accounting for better results than the previous choices, such as photodynamic therapy (PDT) and laser photocoagulation. Currently, three drugs – bevacizumab, ranibizumab, and aflibercept work well, so as to achieve a rapid resolution of exudative signs in most patients [**10**].

However, the first option was pegaptanib sodium, a selective VEGF isoform 165 inhibitor, approved by the FDA in 2004 for the treatment of neovascular AMD. Although the VISION study proved its therapeutic benefit, which was better than PDT, visual acuity remained low and it was soon exceeded by the next anti VEGF, ranibizumab. Therefore, pegaptanib is no longer recommended in the treatment of wet AMD [**4**].

Bevacizumab is a full-length recombinant monoclonal antibody, which binds all isoforms of VEGF, and was approved by the FDA in 2004 for the intravenous treatment of metastatic colorectal cancer. The SANA study showed promising results after two or three bevacizumab intravenous doses, with a mean gain of 14 ETDRS letters at 24 weeks. The first case of intravitreal bevacizumab was reported one year later, with good results after just one month and no adverse effects. It soon became widely used in the treatment of wet AMD, due to its good results, safe profile and reduced cost, but in an OFF LABEL manner [**6**].

Ranibizumab is a monoclonal antibody fragment, with a hundred times higher affinity than bevacizumab, for all VEGF isoforms, approved by the FDA in 2006, for the monthly intravitreal treatment of wet AMD. The MARINA study compared it to sham injections, with positive results: patients gained a mean 6.6 ETDRS letters after 2 years, compared to a mean loss of 14.9 ETDRS letters in the sham group. The ANCHOR study compared intravitreal ranibizumab to PDT. At one year, the mean gain in the first group was 11.3 ETDRS letters, while the PDT group lost a mean 9.5 letters. Given the similarities between bevacizumab and ranibizumab, in the matter of structure, results and side effects, and the significant price difference (50 $ vs. 2000 $ per injection), the CATT study was initiated, aiming to compare the two substances. There was no significant difference both at one and two years, between monthly bevacizumab and ranibizumab (+ 8.5 vs. + 8.0 ETDRS letters mean visual gain and 196 vs. 168 micron decrease in central retinal thickness at one year) or as needed – which will be discussed later – bevacizumab and ranibizumab. Also, the GEFAL and IVAN studies found similar results at one and two years respectively. The CATT and IVAN studies showed no statistical significant differences in the local side effects, such as endophthalmitis, uveitis, retinal detachment, vitreous hemorrhage, traumatic cataract, and systemic side effects, such as atherothrombotic events between the two drugs. However, there have been more frequent gastrointestinal hemorrhages and infections among patients treated with bevacizumab [**11**].

Aflibercept, the newest treatment option, is a fusion protein, with a high affinity for VEGF-A, VEGF-B and PlGF (placental growth factor), approved by the FDA in 2012, for the treatment of wet AMD, in a bimonthly regimen, after a loading phase of three monthly doses. The VIEW 1 and 2 studies proved that bimonthly 2 mg aflibercept is non-inferior to monthly 0.5 mg ranibizumab, with similar ocular and systemic adverse events, and a slightly lower cost. Aflibercept has also shown positive results in non-responders, previously treated with bevacizumab or ranibizumab [**11**].

Taking into consideration that ranibizumab is approved and recommended for monthly intravitreal injections for long periods of time, its price tag is high, and the number of patients is constantly increasing, the burden on both the health care systems and the patients is extremely high. Therefore, we began asking ourselves whether similar results could be obtained, with fewer injections needed for each patient. Thus, we could lower the costs, the risks, the natural evolution towards geographical atrophy, and avoid over-treating patients [**5**].

The first option that was taken into account was the bimonthly treatment. However, the PIER and EXCITE studies provided modest results. In the PIER study, patients underwent a loading phase of 3 monthly ranibizumab injections, followed by a quarterly retreatment, compared to sham injections. At 24 months, patients experienced a mean loss of 2.2 ETDRS letters from baseline and a significant decrease in the best-corrected visual acuity (BCVA) achieved during the loading phase. The EXCITE study compared monthly to quarterly ranibizumab, after a loading phase. The difference between the two groups at month 12 was 4.5 ETDRS letters, with morphological differences noticeable on OCT. Therefore, the quarterly treatment was abandoned due to its poor results, both anatomic and functional [**8**].

Another less frequent treatment regimen is the PRO RE NATA (PRN), or Treat and Observe. It consists of a three monthly injection loading phase, followed by a monthly follow-up and retreatment as needed. The retreatment criteria include visual acuity loss without other reasons, hemorrhage, or edema upon fundus examination, leakage on fluorescein angiography, or increased central retinal thickness, due to intra or subretinal fluid, on OCT examination [**7**]. The PRN regimen was first investigated in the small PrONTO study. During the two years of follow-up, the 37 patients were assessed on a monthly basis, and received intravitreal 0.5 mg ranibizumab injections whenever a mean visual acuity loss of 5 ETDRS letters or a 100-micron increase in the central retinal thickness were noted. The visual results were very good, comparable with those of the pivotal studies, ANCHOR and MARINA: the mean gain was + 11 ETDRS letters, compared to baseline, achieved with far fewer injections: 9.9 over two years [**10**]. The SUSTAIN study examined the efficacy and safety of ranibizumab administered to 513 patients on a PRN basis, following three monthly loading doses. Retreatment criteria were the same as in the PrONTO study; BCVA increased during the 12 months of follow-up, with a mean gain of 3.6 letters from baseline, and a mean 5.6 injections administered. However, most of the visual increase was achieved during the loading phase, followed by a slight decrease afterwards [**8**]. The results of the larger SAILOR study (n = 2378) were not as favorable, with a slight decline in the visual acuity over time, due to the quarterly visit protocol. Therefore, patients were probably undertreated [**10**]. HORIZON is a two-year extension study, following patients who had completed the ANCHOR, MARINA, or FOCUS trials, in order to evaluate the long term safety and efficacy of intravitreal 0.5 mg ranibizumab as needed. The results showed a decline in BCVA gains achieved with a previous monthly treatment, thus highlighting the strict need for a continuous follow-up and rigorous retreatment criteria. The best results achieved on a PRN regimen were obtained in the HARBOR study, which is the only one to include the SD- OCT monitoring. Participants (n = 1098) received 0.5 or 2 mg ranibizumab, monthly vs. as needed, after three monthly loading doses. At 12 months, results showed no significant difference between the 0.5 and 2 mg groups, while at 24 months, the mean change in BCVA from baseline was + 9.1 letters in the monthly 0.5 mg injection group, compared to + 7.9 letters in the 0.5 mg PRN group, which received a mean 13.3 treatments during the two years period. Therefore, this study confirmed that the monthly 0.5 mg ranibizumab provided optimum results, while there was no significant disadvantage in using a PRN protocol, provided that patients were strictly monitored, using SD-OCT technology [**11**]. Back to the CATT study, data specific for year two showed a mean difference between the monthly and PRN ranibizumab groups of only 2.4 letters and 29 microns in the central retinal thickness, with a mean 22-23 monthly injections, compared to a significantly lower 12-14 in the PRN group. Also, more geographic atrophy cases were reported in the monthly treatment group [**8**].

Finally, the Treat and Extend (TAE) protocol is the most recent less frequent regimen, gaining more and more popularity; about 78% of the American retina specialists reportedly used this approach in 2013. Patients are treated with monthly injections, until no signs of choroidal neovascularization (CNV) activity are observed on the slit lamp examination and OCT. Signs of disease activity include intra, subretinal fluid or hemorrhages in the macula. Follow-ups are then extended by two-week intervals as long as no CNV activity is detected. However, if exudation is present, treatment intervals are shortened by 2 weeks. Thus, the anti VEGF treatment is administered at each visit, regardless of the disease activity, but the increasing intervals between visits allow fewer injections [**9**]. The advantages of this regimen are decreased costs, fewer visits and therefore reduced burdens for both patients and physicians. Also, given the fact that therapeutic VEGF suppression varies among patients, between 26 and 69 days, with a maximum at 36–38 days, the TAE protocol manages to blend with each patient’s response pattern. The TAE protocol also seeks to reduce macular hemorrhages, which are sometimes reported in PRN treated patients, sometimes untreated for longer periods of time [**8**]. Spaide and Freund were the first to describe a TAE regimen with good results. A few patients were followed for three years, averaging 7 ranibizumab treatments yearly and a mean visual improvement of 4 lines. Oubraham et al. reported better results with the TAE regimen, compared to PRN: +10.8 vs. +2.3 ETDRS letters at 12 months, achieved with 7.8 and 5.2 injections, respectively. Gupta et al. compared bevacizumab to ranibizumab on a TAE regimen, with similar results at 18 months. The LUCAS study also compared bevacizumab to ranibizumab on a TAE basis, with no loading phase. The results at one year were found to be equivalent, with a mean gain of 8.0 and 8.2 letters respectively, and a similar number of visits. Freund et al. found that the mean interval between retreatments settled at around 10 weeks [**6**]. The largest recent study documented was the 3 year treatment outcomes for 196 patients with neovascular AMD, treated with bevacizumab or ranibizumab on a TAE regimen. On average, the patients received 17 injections over the 3-year period; participants gained a mean 13.6 ETDRS letters, the central retinal thickness decreased by about 75 microns, irrespective of the drug used, as measured on SD-OCT, and the mean interval between visits was 13 weeks. Also, the visits were 50% less than in PRN studies [**9**]. Another recent study compared 0.5 mg ranibizumab administered to 60 patients in a TAE vs. TAO regimen, during a three year period. There were, however, no significant differences among the visual activity outcomes, central retinal thickness (as measured by SD-OCT) and the number of injections between the two treatment strategies [**2**].

While debates continue over which treatment protocol provides the best results, new substances are being developed, which will probably change the way these patients are managed. Fovista®, a Platelet Derived Growth Factor inhibitor, binds pericytes on new vessels, thus inhibiting their growth; conbercept promises similar results to aflibercept’s, but at a lower price; squalamine and pazopanib eye drops are currently in phase II trials [**3**].

To sum up, anti VEGF therapy has removed wet AMD from the list of incurable diseases and the optimum treatment choice should provide a reasonable balance between cost and benefit. Although treat and extend seems to provide the most effective results, there is still insufficient evidence to determine the best treatment option and more studies are needed to compare protocols. However, there are still unanswered questions, such as the following: do we need an individualized treatment protocol for aflibercept? And what is to be done with non responders? Regardless, the best results are only achieved with early, correct diagnosis and treatment initiation, followed by strict follow-up.

## References

[1] (2015). American Academy of Ophthalmology Retina/ Vitreous Panel. Preferred Practice Pattern Guidelines. Age- Related Macular Degeneration.

[2] Calvo P, Wang Y, Ferreras A, Lam WC, Devenyi R (2014). Treat and Extend Versus Treat and Observe Regimens in Wet Age-related Macular Degeneration Patients Treated with Ranibizumab: 3-year Surveillance Period. J Clin Exp Ophthalmol.

[3] Danis R, McLaughlin MM, Tolentino M, Staurenghi G, Ye L, Xu CF, Kim RY, Johnson MW (2014). Pazopanib Eye Drops: A Randomised Trial in Neovascular Age related Macular Degeneration. Br J Ophthalmol.

[4] Holz FG (2014). Recent developments in the treatment of age-related macular degeneration. J Clin Invest.

[5] Holz FG, Korobelnik JF, Lanzetta P, Mitchell P, Schmidt- Erfurth U, Wolf S, Markabi S, Schmidli H, Weichselberger A (2010). The Effects of a Flexible Visual Acuity–Driven Ranibizumab Treatment Regimen in Age-Related Macular Degeneration: Outcomes of a Drug and Disease Model. Invest Ophthalmol Vis Sci.

[6] Kovach JL, Schwartz SG, Flynn HW Jr., Scott IU (2012). Anti- VEGF Treatment Strategies for Wet AMD. Journal of Ophthalmology.

[7] Lalwani GA, Rosenfeld PJ, Fung AE, Dubovy SR, Michels S, Feuer W, Davis JL, Flynn HW Jr, Esquiabro M (2009). A variable-dosing regimen with intravitreal ranibizumab for neovascular age-related macular degeneration: year 2 of the PrONTO Study. Am J Ophthalmol.

[8] Lanzetta P, Mitchell P, Wolf S, Veritti D (2013). Different antivascular endothelial growth factor treatments and regimens and their outcomes in neovascular age- related macular degeneration: a literature review. Br J Ophthalmol.

[9] Rayess N, Houston SK 3rd, Gupta OP, Ho AC, Regillo CD (2015). Treatment outcomes after 3 years in neovascular age- related macular degeneration using a treat-and-extend regimen. Am J Ophthalmol.

[10] Regillo CD (2014). Anti-VEGF maintenance therapy for neovascular AMD. Retina Today.

[11] Schmidt-Erfurth U (2014). Br J Ophthalmol.

